# Endoscopic ultrasound with submucosal saline injection improves the accuracy of T1a and T1b staging in superficial esophageal squamous cell carcinoma

**DOI:** 10.3389/fmed.2025.1509628

**Published:** 2025-04-30

**Authors:** Jianjun Zhang, Ming Chen, Yongsheng Gao, Jinqi Liu, Zengjun Li, Dongyang Wang

**Affiliations:** ^1^Department of Oncology, Shandong Cancer Hospital and Institute, Shandong First Medical University and Shandong Academy of Medical Sciences, Jinan, China; ^2^Department of Endoscopy, Shandong Cancer Hospital and Institute, Shandong First Medical University and Shandong Academy of Medical Sciences, Jinan, China; ^3^Department of Pathology, Shandong Cancer Hospital and Institute, Shandong First Medical University and Shandong Academy of Medical Sciences, Jinan, China

**Keywords:** endoscopic ultrasonography, superficial esophageal squamous cell carcinoma, submucosal saline injection, tumour stage, treatment options

## Abstract

**Background:**

Endoscopic ultrasound (EUS) is important for diagnosing and staging esophageal cancer. However, substantial variability in the diagnostic and staging accuracy of EUS, especially in early-stage cancers, affects patients’ treatment choices and quality of life.

**Aims:**

To explore whether conventional endoscopic ultrasonography (EUS-C) combined with submucosal saline injection (EUS-SSI) improves diagnostic accuracy in preoperative T1a and T1b staging in superficial esophageal squamous cell carcinoma (SESCC).

**Methods:**

Patients with SESCC first underwent EUS-C. Then, they received SSI and underwent a repeat EUS. The diagnostic accuracy of EUS-C and EUS-SSI was evaluated based on the final postoperative pathology results.

**Results:**

A total of 92 patients with endoscopically diagnosed SESCC were included in the study. Postoperative pathology confirmed superficial SESCC in all patients (T1a stage, *n* = 77; T1b stage, *n* = 15). EUS-C correctly identified 54 of 77 patients with T1a cancer and nine of 15 patients with T1b cancer, whereas EUS-SSI identified 68 of 77 patients with T1a cancer and 10 of 15 patients with T1b cancer. EUS-SSI was more accurate than EUS-C in diagnosing T1a and T1b stage SESCC (84.8 and 68.5%, respectively).

**Conclusion:**

EUS-SSI differentiates between T1a and T1b stages of superficial SESCC with better diagnostic accuracy than EUS-C, thereby reducing the rate of over-staging.

## 1 Introduction

Superficial esophageal squamous cell carcinoma (SESCC) is a malignant lesion confined to the mucosa or submucosa regardless of the presence or absence of lymph node metastasis (1, 2). Endoscopic submucosal dissection (ESD) is widely used to treat SESCC (3). Indications for ESD have been expanded to cases in which the risk of lymph node metastasis is assumed to be low. Even if the pathological invasion depth is classified as T1b (tumour invasion of the submucosa), ESD can be performed if the invasion is limited to SM1 (submucosal invasion <200 μm from the muscularis mucosa) (4–6), and it is not suitable for submucosal invasion ≥200 μm (7–9).

Therefore, accurate determination of the depth of invasion (T stage) of esophageal cancer is crucial in determining treatment strategies.

Conventional endoscopic ultrasonography (EUS-C) is a technique that can provide clear cross-sectional images of the gastrointestinal (GI) tract wall. It helps diagnose the depth of submucosal tumours or assess cancer invasion, which is why it has been used for T staging of esophageal cancer (10, 11). Although previous studies have shown the clinical efficacy of EUS in T staging of esophageal cancer (12), the results have been largely variable (13). EUS-C cannot satisfactorily distinguish between T1a and T1b stages of esophageal squamous cell carcinoma (11, 14). Specifically, it is difficult to distinguish between T1a (tumour invading the lamina propria and muscularis mucosae) and T1b lesions by EUS because of the thin border between the mucosa and submucosa and overlap in echogenicity (15). Therefore, it is necessary to develop reliable and less invasive methods to distinguish between the T1a and T1b stages of SESCC.

Submucosal saline injection (SSI) is a necessary step in the ESD treatment of early esophageal cancer (16). SSI can increase the thickness of the submucosal layer to prevent gastrointestinal perforation caused by damage to the muscularis propria during treatment (17). And physiological saline is a good transmission medium for sound waves and can be used as an echo contrast enhancer for ultrasound transmission (18, 19).

Therefore, we evaluated whether SSI can be used to improve the accuracy of EUS in distinguishing between T1a and T1b stages of SESCC and analyse potential factors that interfere with staging results.

## 2 Methods

### 2.1 Study population

In 92 patients with endoscopically diagnosed SCC, EUS was performed on 92 lesions. All patients underwent EUS-C followed by EUS with SSI (EUS-SSI).

The tumour morphology under endoscopic evaluation was classified according to the Paris endoscopic classification for superficial neoplastic lesions (20) as follows: type I (protruded), type 0–IIa (superficial elevated), type 0–IIb (flat), type 0–IIc (superficial depressed), and type III (excavated).

### 2.2 Procedure and equipment

**Figure 1:**
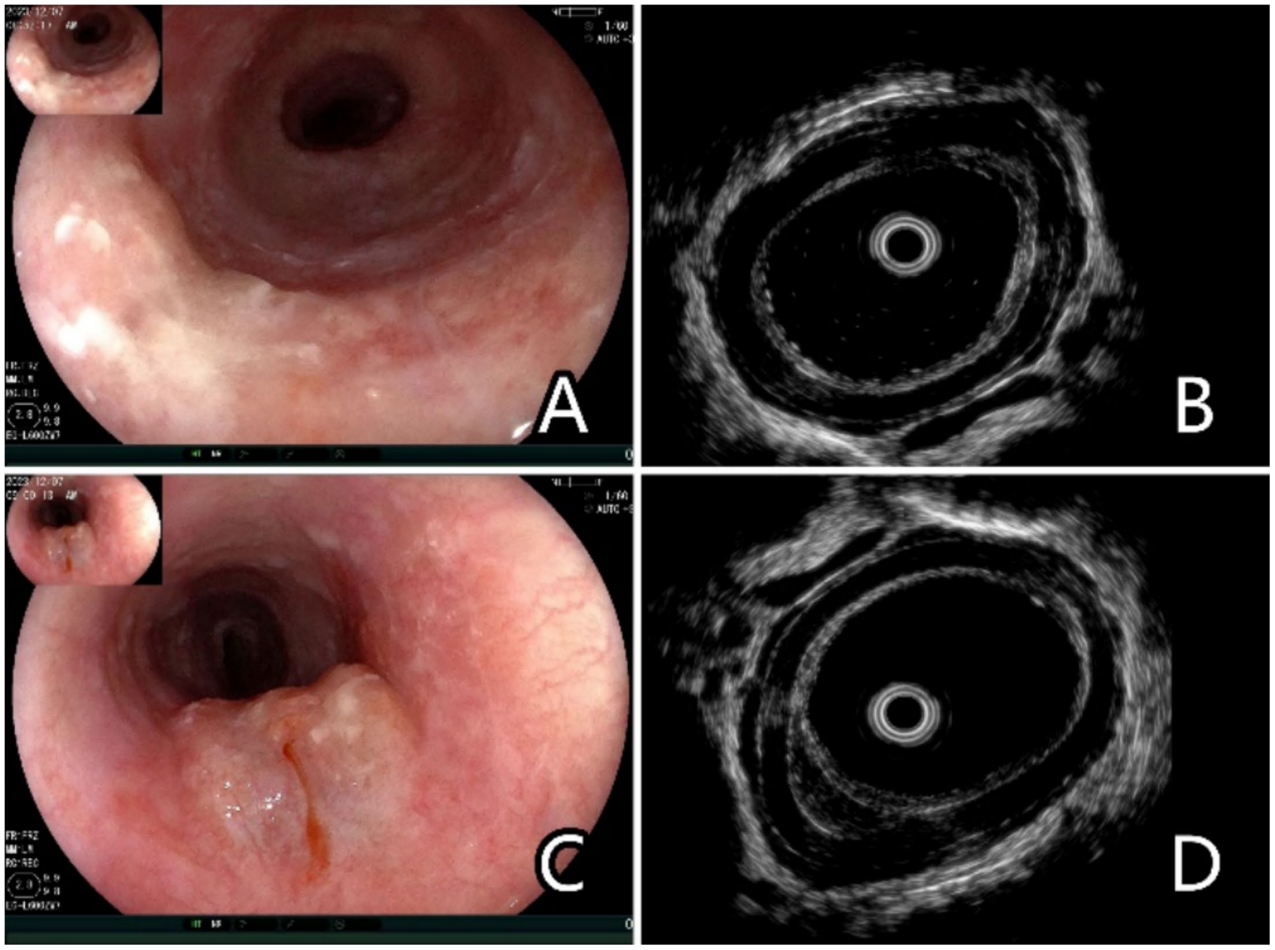
Endoscopic and ultrasonographic images and associated schematic diagrams of T1a superficial esophageal squamous cell carcinoma. **(A)** Lesions found in the middle oesophagus under white light endoscopy, 0–IIb + IIa, 2 cm × 2 cm. **(B)** Conventional ultrasonography (EUS-C) revealed that the mucosal layer was thickened and hypoechoic, and it is difficult to differentiate the extent of invasion from the mucosal layer to the submucosal layer. **(C)** Saline injection can facilitate the lifting of the lesions. **(D)** Ultrasonography after saline injection (EUS-SSI) shows that the boundary between the mucosa and submucosa is clearly displayed, suggesting that lesions with depth of infiltration limited to the mucosal layer can be easily identified, and the submucosa can be clearly distinguished from the mucosa.

The SSI protocol involved the injection of saline using a 23G disposable mucosal needle (NM-400 U, Olympus). A 20 MHz ultrasonic probe was used for the EUS examination (P2620-M, Fujifilm, Japan). Around 3–5 mL of normal saline was injected into the submucosa. The puncture point was located 0.5 cm outside the edge of the lesion. The saline injection was stopped when the esophageal mucosa rose by approximately 1 cm. After SSI, the examiner used EUS to determine the depth of the lesion (Figure 1).

### 2.3 Outcome assessment

T1a superficial SESCC on EUS was determined based on low-echoic lines of muscularis mucosae that were clearly demarcated from the submucosa. T1b superficial SESCC was determined based on low-echoic line lesions that were not clearly distinguished from the boundary of the submucosal layer. Subsequently, the patients underwent endoscopic or surgical resection within 7 days.

EUS examination and staging were performed simultaneously and were completed by two senior physicians with at least 3 years of EUS experience.

All recruited patients agreed to be enrolled as participants in this clinical trial and provided their informed consent. This study was approved by the Ethics Institutional Review Committee of Cancer Hospital Affiliated with Shandong First Medical University.

### 2.4 Statistical analysis

All statistical analyses were conducted using SPSS version 22.0 software (SPSS Inc., Chicago, IL). The chi-square test or Fisher’s exact test was used to compare the baseline characteristics of two groups divided by EUS result. In each groups, continuous variables were given as median and interquartile range and categorical variables were given as number and percentage. Predictors associated with T overstaging by EUS in pathologic in the univariable analysis were included in the multivariable logistic regression analysis and probability value less than 0.05 was considered significant.

## 3 Results

### 3.1 Baseline characteristics and T staging based on postoperative pathological parameters

All patients showed good tolerance to the endoscopy procedure. There were no adverse events such as serious bleeding, choking, esophageal wall perforation, or anaesthesia-related problems.

The postoperative pathology of 92 patients showed that they all had T1 superficial SESCC (T1a in 77 cases and T1b in 15 cases). No stage T2–T4 tumours were observed. There were no cases of positive lymph nodes assessed via enhanced computed tomography or EUS with or without SSI and postoperative pathology (Table 1).

**Table 1:** Clinical features and postoperative pathological results of 92 patients with superficial esophageal squamous cell carcinoma.

Variables	Date	*p*-value
Age (group)		*p* = 0.531
≤64 years^a^	43	
>64 years	49	
Sex		*p* < 0.01
M	81	
F	11	
Final pathology		*p* < 0.01
T1a	77	
T1b	15	
T1b-SM1	8	
T1b-SM deep	7	
Differentiation		*p* = 0.003
High-grade dysplasia	8	
Well	39	
Moderately	31	
Poor	14	
Site of neoplasm		*p* < 0.01
Upper third	9	
Middle third	50	
Lower third	29	
Endoscopic morphology (EGC type)		*p* < 0.01
0–IIa	11	
0–IIb	66	
0–IIc	15	
Size (mm)		*p* < 0.01
≤20 mm	43	
>20 mm, <50 mm	36	
≥50 mm	13	

M, male; F, female; T1b-SM1, depth of submucosal invasion <200 μm; T1b-SM deep, depth of submucosal invasion DA ≥200 μm.

a Median age.

### 3.2 Comparison of T staging outcomes between the EUS-C and EUS-SSI

EUS-C identified 54 of 77 patients with T1a cancer, whereas EUS-SSI identified 68 of 77 patients with T1a cancer, and EUS-C identified nine of 15 patients with T1b cancer, EUS-SSI identified 10 of 15 patients with T1b cancer. In the present study, EUS-SSI was more accurate than EUS-C in diagnosing T1a and T1b lesions of EGC (84.8 and 68.5%, respectively) (Table 2).

**Table 2:** Preoperative and postoperative staging results for superficial esophageal squamous cell carcinoma with conventional endoscopic ultrasonography and endoscopic ultrasonography after submucosal saline injection.

		Postoperative pathology stage	
Postoperative EUS reported stage		T1a^a^	T1b
EUS-C, *n* (%)			
	T1a	54 (70.1%)^b^	6 (40%)
	T1b	23 (29.9%)	9 (60%)
EUS-SSI, *n* (%)			
	T1a	68 (88.3%)^b^	5 (33.3%)
	T1b	9 (11.7%)	10 (66.7%)

a As preplanned, cases with high-grade dysplasia were classified into T1a; EUS, endoscopic ultrasonography; SSI, submucosal saline injection.

b The proportion of T1a detected by EUS-SSI VS. EUS-C (88.3% vs. 70.1%, *p* < 0.05).

### 3.3 Comparison of over-staging rate between the EUS-C and EUS-SSI

T1b stage lesions were diagnosed by EUS-C in 32 cases preoperatively, but postoperative pathology results suggested that these were cases of T1a (*n* = 15) and T1b-SM1 (depth of submucosal invasion <200 μm; *n* = 8) cancers. T1b stage lesions were diagnosed by EUS-SSI in nine cases preoperatively, but postoperative pathology results suggested that these were cases of T1a (*n* = 4) and T1b-SM1 (*n* = 5) cancers. The over-staging rate of EUS-SSI was lower than that of EUS-C (9.8 and 25.0%, respectively) (Table 3).

**Table 3:** Rate of misdiagnosis for T staging of superficial esophageal squamous cell carcinoma with endoscopic ultrasonography after submucosal saline injection.

Group	EUS-C	EUS-SSI	*p*-value
Overstaging, *n* (%)	23 (25.0%)	9 (9.8%)	*p* < 0.01
Understaging, *n* (%)	6 (6.5%)	5 (5.4%)	*p* = 0.77

EUS, endoscopic ultrasonography; SSI, submucosal saline injection.

### 3.4 Analysis of risk factors for over-staging based on EUS-SSI

Among the nine patients who were over-staged by EUS-SSI, endoscopic gross classification included one case of 0–IIa, six cases of 0–IIb, and two cases of 0–IIc. Preoperatively, six patients underwent multiple endoscopic biopsy sections in different hospitals. Scars formed by multiple biopsies were seen in the lesion area. The postoperative pathological results showed poorly differentiated SCC in four lesions (Table 4).

**Table 4:** Clinical features and endoscopic ultrasonography findings before and after submucosal saline injection, and pathological results of nine patients with superficial esophageal squamous cell carcinoma.

No.	Age	Sex	Site	Endoscopy morphology (EGC type)	Size (max, mm)	Biopsies times	Number of biopsies	EUS-C stage	EUS-SSI stage	Pathology stage	Differentiation
1	59	M	Upper third	0–IIa	35	4	6	T1b	T1b	T1b-SM1	Poor
2	62	M	Upper third	0–IIb	65	3	5	T1a	T1b	T1b-SM1	Moderately
3	57	F	Lower third	0–IIb	55	3	5	T1b	T1b	T1b-SM1	Poorly
4	59	M	Middle third	0–IIc	25	3	5	T1a	T1b	T1a	Well
5	63	M	Middle third	0–IIb	45	3	4	T1b	T1b	T1a	Poor
6	66	M	Upper third	0–IIc	55	3	4	T1a	T1b	T1b-SM1	Moderately
7	67	F	Lower third	0–IIb	15	2	3	T1b	T1b	T1a	Moderately
8	79	F	Middle third	0–IIb	60	2	3	T1a	T1b	T1b-SM1	Poor
9	74	M	Upper third	0–IIb	20	2	3	T1a	T1b	T1a	Poor

M, male; F, female; T1b-SM1, depth of submucosal invasion <200 μm; T1b-SM deep, depth of submucosal invasion DA ≥200 μm.

## 4 Discussion

Accurate staging is an important basis for selecting treatment options for esophageal cancer (21). Patients with SESCC diagnosed at early stages have a higher survival rate than those diagnosed at advanced stages (22).

With the development of high-definition endoscopy and endoscopic staining technology, the detection rate of early esophageal cancer continues to increase, which is contributing to reducing the mortality rate associated with esophageal cancer (23). Radical esophagectomy and super-minimally invasive resection under digestive endoscopy are the main treatments for early-stage esophageal cancer (24–26). However, the choice of treatment depends on the staging, i.e., T1a or T1b (25, 26). Surgery is usually recommended for T1b cancer cases, whereas ESD is an option for T1a cancer cases (24, 26). Unlike radical esophagectomy, ESD preserves the integrity of organs and physiological structures, and patients have a better quality of life after treatment (27, 28). Therefore, it is crucial to accurately distinguish between T1a and T1b, and EUS is the preferred method for staging.

Although EUS has been used to assess the depth of invasion in early esophageal cancer cases, the reported diagnostic accuracy ranges from 60 to 80% (11, 14). The staging accuracy of EUS-C by two experienced endoscopists was 68.5% in our study, which is consistent with previous reports. However, after SSI, the accuracy of EUS staging for early-stage SESCC increased to 84.8%, suggesting improved staging accuracy for early-stage esophageal cancer.

Some studies have postulated that EUS has no significant advantage over traditional endoscopy and magnification endoscopy in predicting the depth of invasion of early esophageal cancer (29).

The accuracy of EUS is reported to vary greatly depending on the experience of the endoscopist, tumour location, macroscopic type of tumour, tumour size, presence or absence of ulceration, and tumour differentiation type (11).

Lesions with severe inflammation or ulceration or in cases where multiple biopsies have been performed, there is evidence of submucosal fibrosis, and hypoechoic lesions observed on EUS resemble tumour infiltration. For lesions in the upper third of the oesophagus, the accuracy of EUS may be reduced due to differences in esophageal wall thickness, poor esophageal lumen relaxation, and air interference. Furthermore, due to the tubular structure of the oesophagus and frequent peristalsis, it is difficult to fill the oesophagus with degassed water and position the EUS probe near the lesion. In addition, larger tumour size is a risk factor for incorrect judgment of invasion depth (30). Undifferentiated tumour cells may have the potential to undergo micro-infiltration in the submucosa of the esophageal wall, and EUS cannot visualize these micro-invasions, thereby underestimating tumour staging (31).

In this study, 15 patients with stage T1a cancer were over-staged as T1b in EUS-C examination. Although it was recommended that patients choose diagnostic ESD treatment, some patients received traditional surgical treatment, and their postoperative quality of life was significantly reduced. Subsequent EUS-SSI staging significantly reduced over-staging (25.0% with EUS-C to 9.8% with EUS-SSI). However, there were four patients whose preoperative and postoperative pathologies were incompatible, ranging from T1b to T1a. Further analysis showed that the lesion was located in the upper one-third of the oesophagus. Multiple preoperative endoscopic examinations and moderate-to-poor differentiation are risk factors for EUS-SSI over-staging. Multiple biopsies under endoscopy lead to scar-like hyperplasia and adhesion between the mucosa and submucosa. With EUS visualization, the boundaries can be unclear and hypoechoic changes between the mucosa and submucosa may be observed. These findings affect the judgment of the invasion depth.

Limitations of EUS-SSI include a longer examination time than EUS-C and the possibility of greater discomfort to the patient. These drawbacks can be addressed by using sedatives.

All enrolled patients underwent esophagectomy or ESD within 1 week, and all recovered well. There was no difficulty in endoscopy dissection due to poor submucosal injection lift. All lesions were completely removed. SSI may elicit an inflammatory response and cause fibroplasia in the submucosa, but it is unknown whether SSI makes ESD treatment more difficult if the patient cannot receive treatment soon. More cases and longer follow-ups are needed for further confirmation.

In this study, submucosal injection improved the accuracy of EUS staging of early-stage, i.e., T1a and T1b stages of SESCC. Lesions located in the upper oesophagus and multiple repeated biopsies are risk factors for inaccurate staging. This study has limitations, including its single-center, non-randomized design, which may introduce selection bias and confounding factors. The small sample size might also limit statistical power. Future multicenter RCTs with larger samples are needed to further validate the advantages of EUS-SSI, incorporating broader outcome measures for a more robust evaluation.

## Data Availability

The raw data supporting the conclusions of this article will be made available by the authors, without undue reservation.
